# Potential of bone morphogenetic protein-7 in treatment of lupus nephritis: addressing the hurdles to implementation

**DOI:** 10.1007/s10787-023-01321-x

**Published:** 2023-08-25

**Authors:** Carine Smith, Riette du Toit, Tracey Ollewagen

**Affiliations:** 1 Experimental Medicine Research Group, Department Medicine, Faculty of Medicine and Health Sciences, Stellenbosch University, Parow, South Africa; 2 Division Rheumatology, Department Medicine, Faculty of Medicine and Health Sciences, Stellenbosch University, Parow, South Africa

**Keywords:** Systemic lupus erythematosus, Tubulo-interstitial inflammation, Fibrosis, TGF-beta, Africa

## Abstract

Up to 50% of systemic lupus erythematosus (SLE) patients world-wide develop lupus nephritis (LN). In low to middle income countries and in particular in sub-Saharan Africa, where SLE is prevalent with a more aggressive course, LN and end stage renal disease is a major cause of mortality. While developed countries have the funding to invest in SLE and LN research, patients of African descent are often underrepresented in clinical trials. Thus, the complex influence of ethnicity and genetic background on outcome of LN and SLE as a whole, is not fully understood. Several pathophysiological mechanisms including major role players driving LN have been identified. A large body of literature suggest that prevention of fibrosis—which contributes to chronicity of LN—may significantly improve long-term prognosis. Bone morphogenetic protein-7 (BMP-7) was first identified as a therapeutic option in this context decades ago and evidence of its benefit in various conditions, including LN, is ever-increasing. Despite these facts, BMP-7 is not being implemented as therapy in the context of renal disease. With this review, we briefly summarise current understanding of LN pathology and discuss the evidence in support of therapeutic potential of BMP-7 in this context. Lastly, we address the obstacles that need to be overcome, before BMP-7 may become available as LN treatment.

## Introduction

Systemic lupus erythematosus (SLE) is an incurable, autoimmune disease of unknown aetiology, which severely limits quality of life and economic productivity in patients (Sutanto et al. 2013; Daly et al. 2015; Rees et al. 2017). SLE has a strong female bias (female to male ratio of ≈ 9:1) and is more prevalent and severe in vulnerable and disadvantaged populations than in Caucasians (Ferucci et al. 2014; Somers et al. 2014; Plantinga et al. 2016; González et al. 2022; Aguirre et al. 2023). More specifically, SLE tends to exhibit higher disease activity and greater damage accrual in non-Caucasian populations (Hispanics, African descendants and Asians). In these populations, SLE disease onset typically occurs at an earlier age (Somers et al. 2014), with a disease duration of only 3-11 years (Greenstein et al. 2019).

More specifically related to lupus nephritis (LN), a large multinational study reported LN to occur in up to 50% of SLE patients in first world countries, with much higher incidence in populations of African descent (Hanly et al. 2015). Together with infections, end stage renal disease (ESRD) is a major cause of mortality in SLE (Lewis and Jawad 2017; Greenstein et al. 2019; Barber et al. 2021), even at a young age. In support of this, 13% of a South African-based pediatric SLE cohort was reported to have ESRD and to need more frequent dialysis than a comparative US cohort (Lewandowski et al. 2017). Furthermore, another South African study reported co-morbid incidence of LN in 67.9% of SLE patients presenting with myocarditis (Du Toit et al. 2017), highlighting the fact that SLE complications commonly involve more than one organ system in these patients. In South African SLE patients with LN, a 5-year survival rate of only 60% was reported in 2007 (Wadee et al. 2007) and 67% in 2017 (Brijlal et al. 2017), significantly lower than that of a predominantly Caucasian population in the United States of America (89% at 5 years) (Hocaoǧlu et al. 2023).

The specific factors responsible for the ethnic disparity in SLE and LN are complex and include genetic, cultural and socioeconomic factors (e.g. geographical place of residence: urban vs rural) (González et al. 2022). Both genetic and non-genetic components influence the expression and outcome of SLE, including disease activity, damage accrual, work disability and mortality. Considering the genetic factors, a distinct SLE-associated auto-antibody profile was reported in African-American SLE patients when compared to European- or Hispanic-American counterparts (Ko et al. 2012). This may explain the relatively poorer response reported in African-Americans with LN, to the drug cyclophosphamide (Dooley et al. 1997), which inhibits T and B lymphocyte proliferation and is currently recommended as one of two options for first line treatment of LN, at least in developed countries (Takada et al. 2001; Fanouriakis et al. 2020). The other first line option for LN—mycophenolate mofetil—seems to yield better results in African patients (Isenberg et al. 2010), but the higher cost of this option makes it relatively less accessible in developing countries. Clearly, more cost-effective treatments are urgently required to bridge this gap.

Despite clear discrepancies in treatment response, ethnic minority groups remain underrepresented in clinical trials (Falasinnu et al. 2018). Other factors, such as patient numbers in developed countries and relatively short durations of clinical trials to date, add to the relatively slow progress in biological drug development for treatment of SLE and specifically LN, when compared to other rheumatic diseases such as rheumatoid arthritis (RA) and ankylosing spondylitis (Lorenzo-Vizcaya and Isenberg 2022). The high cost of genetic profiling and individualized medicine is another significant obstacle to drug development progress in the poorer populations most burdened by SLE.

Given the lack of potential treatments for LN in low to middle income countries (LMICs) such as sub-Saharan Africa in particular—where the majority of SLE patients are treated with glucocorticoids and the repurposed anti-malarial drug hydroxychloroquine only (Okpechi et al. 2015; Jorge et al. 2021)—identification of novel treatments aimed at novel therapeutic targets is clearly a priority. Here, we firstly review the pathohistological profile of LN to identify major therapeutic targets. We then review the potential of bone morphogenetic factor-7 (BMP-7) as a LN treatment, by reviewing relevant mechanistic studies from both rheumatic and non-rheumatic literature. Importantly, we focus on the unique ability of BMP-7 to modulate cellular communication at tissue level in the context of LN. Thereafter we present recommendations on how a more beneficial BMP-7 activity profile may potentially be achieved therapeutically. Lastly, we present the complexities potentially hampering the development of BMP-7 as a treatment intervention.

## Pathohistological profile of lupus nephritis

LN is clinically identified through urinary abnormalities including proteinuria and/or an active urinary sediment. Thereafter, renal biopsies are fundamental in the classification and management of LN (Gasparotto et al. 2020). Classification of LN pathology is largely centered around glomerular pathology. Categories include mesangial LN, where mesangial immune complex deposition is evident (Class I and II), focal (Class III) or diffuse (Class IV) LN where proliferative glomerular lesions occur, or membranous LN (Class V) when subepithelial immune complexes are observed. End stage LN (Class VI) is characterized by large scale global glomerulosclerosis (Chang et al. 2021). Other classifications also exist, such as the National Institute of Health (NIH) activity and chronicity indices, where the activity index points to ongoing renal injury which may respond to treatment, while the chronicity index quantify irreversible damage such as scarring and fibrosis.

Despite heterogeneity and diversity in patient profiles, RNA sequencing identified commonalities in immune cell profiles in kidney biopsies from patients with LN. Here, both resident and infiltrating myeloid cells were present consisting of inflammatory, phagocytic and M2-like cells. In addition to these, T cells, NK cells, a spectrum of activated and naïve B cells, and plasma cells and plasmablasts were present. Furthermore, a clear interferon response was observed in these cells (Arazi et al. 2019). Additionally, podocytes, mesangial cells and epithelial cells contribute to disease development (Tsokos et al. 2016). The deposition of immune complexes into the subendothelium triggers an inflammatory cascade contributing to increased permeability of the endothelium—this impairment is associated with the formation of glomerular lesions (Oates et al. 2022). In terms of specific cellular role players in glomerular damage, immune-complex driven inflammation is commonly considered to be a key role player, since serum levels of anti-double stranded DNA (anti-dsDNA) autoantibodies correlate with extent of glomerulonephritis in LN (Rekvig 2019). Neutrophils—the major contributor to secondary cellular damage in inflammation—are only occasionally observed in glomerulonephritis, and not at all in tubulointerstitial inflammation (TII) (Chang et al. 2021), suggesting that while neutrophils may respond to autoimmune processes, they are unlikely to be the main drivers of LN.

The glomerulus-centered approach to disease classification and monitoring may be an obstacle in itself, since TII and specifically interstitial fibrosis and tubular atrophy (IF/TA), were shown to be better prognostic indicators than glomerular injury in LN (Hsieh et al. 2011), and especially so in populations of African descent (Hsieh et al. 2011; Londoño Jimenez et al. 2018). Furthermore, the markers commonly used to monitor LN progression, which includes anti-dsDNA and low compliment activity, were shown to poorly correlate with TII and IF/TA (Londoño Jimenez et al. 2018).

Of particular interest to our context of tissue level cellular communication, germ cell-like structures with proliferating B cells have been reported to exist within the tubulointerstitium, suggesting tertiary lymphoid neogenesis in LN (Chang et al. 2011). While predictably much research has focused on the characterization and potential amelioration of these B cell-centered processes, the resultant inflammatory and fibrotic sequelae has received much less attention. Nevertheless, the demonstration of in situ dysregulation and tertiary lymphoid neogenesis, suggest focal points of cellular dysregulation. Thus, therapeutic modulation or normalization of the cellular milieu, may significantly impact prognosis.

It is in this context in particular, that we propose a significant potential for BMP-7, formerly known as osteogenic protein-1 (OP-1). However, before unpacking specific cellular mechanisms where BMP-7 may have beneficial effect, we will briefly link BMP-7 to the female predominance in SLE and LN, to point out the importance of consideration of sex and/or the role of estrogens in drug discovery research in the context of female-predominant diseases.

## BMP-7: a link to sexual disparity in LN

Even though potentially its most striking statistic, the female predominance in SLE in general, as well as in LN, has been largely overlooked in drug discovery, with literature focused on the role of estrogen fairly dated. Estrogens have long been established as stimulators of autoimmunity, while androgens have known protective effects in this context (Ortona et al. 2016). Of importance to the current discussion, in addition to direct actions of female hormones in autoimmunity, estrogens may also exert indirect effects. While a reduction of estrogen levels in females is not a viable therapeutic option apart from avoiding its use as hormone replacement therapy or in oral contraceptives (Askanase 2004), it may be possible to manipulate these indirect effects of estrogen for therapeutic outcome.

For example, estrogen has been linked to altered microbiome profile in SLE (Christou et al. 2019), another key contributor to inflammation. Although research into LN management strategies is considering the role of the microbiome, the focus seems to be on changing the constituents of the microbiome to prevent microbe-associated autoantibody formation, as recently reviewed (Pan et al. 2021), leaving a gap to address the sexual disparity in SLE. Potential for an approach to ameliorate effects of estrogen on the microbial secretome (i.e. correcting functional, rather than compositional dysbiosis) has been demonstrated in irritable bowel syndrome, where modulation of the effect of estrogen on microbial secretome resulted in therapeutic benefit in terms of inflammatory outcome and endothelial integrity (Pretorius and Smith 2020, 2023; Pretorius et al. 2022). Despite the central role of inflammation and endothelial damage in LN, this potential therapeutic route remains largely unexplored. In terms of a potential link to BMP-7, a PubMed search revealed only one paper suggesting an interplay of BMP-7 with microbiota: in the context of Langerhans cells—which are sentinel cells known to regulate the balance between inflammation and immune tolerance—BMP-7 was shown to translocate Langerhans cell precursors to the epithelial surface, where transforming growth factor-β (TGF-β) and microbiota action induced its differentiation to ensure mucosal maintenance (Capucha et al. 2018). Although Langerhans cells are not commonly implicated in SLE, this study does suggest functional interplay between BMP-7, TGF-β and microbial role players.

However, strong evidence of a more direct link between BMP-7 and female hormones exists. In this context, a plethora of literature suggest opposing roles for estrogens and BMP-7, which—given the known anti-inflammatory and anti-fibrotic role of BMP-7 (Narasimhulu and Singla 2020; Ollewagen et al. 2022)—may explain the female predominance of not only SLE, but chronic inflammatory conditions in general. For example, already more than two decades ago, estrogen was shown to oppose the BMP-7-induced apoptosis required for tissue remodeling in reproductive tissue (Monroe et al. 2000). Estrogen was subsequently suggested to regulate BMP-7 receptor (ActRII and ALK-2) levels (Shimizu et al. 2006). However, it is possible that the increase in ActRII and ALK-2 gene expression and mRNA levels in the presence of estrogen observed in this study, was a counter mechanism to oppose the effects of estrogen, as a subsequent paper reported BMP-7 to also directly correlate with estrogen receptor (ER) expression changes (Schwalbe et al. 2003) in the context of estrogen-driven breast cancer. This interpretation is supported by the demonstrated BMP-7 inhibition of estrogen-associated breast cancer cell proliferation via a p38 mitogen activated protein kinase pathway (Takahasi et al. 2008). We could not find any information on the potential binding affinity for ER for BMP-7, but such data could shed more light on mechanisms involved.

Of further interest, a more recent study in the context of renal damage elegantly illustrated estrogen to increase the TGF-β/BMP-7 ratio and linked this relatively lower proportion of BMP-7—which opposes the pro-fibrotic effect of TGF-β—to poorer disease outcome (Ziller et al. 2020). This indicates the importance of assessing the TGFβ/BMP-7 ratio, rather than either in isolation, before firm conclusions on the effect of either can be made. Although many gaps still exist in our understanding of the interplay between estrogen and BMP-7, these data support the priority of a BMP-7-centred approach to drug discovery in LN. In the following section, evidence for beneficial mechanistic effects of BMP-7 relevant to LN will be comprehensively reviewed.

## Cellular pathology in lupus nephritis: the BMP-7 link

BMP-7 belongs to the TGF-β superfamily of growth and differentiation factors. Interest in BMP-7 spiked after its demonstrated involvement in osteoblast differentiation and bone formation, and thereafter in renal, bone and eye development (Singla et al. 2012). BMP-7 and TGF-β bind to distinct type II receptors, forming complexes with specific type I serine-threonine kinase (ALK) receptors. This leads to intracellular signaling mediated by Smad proteins (Zeisberg et al. 2003; Daans et al. 2008). BMP-7 is largely expressed in the kidney—specifically in the collecting ducts, distal tubules and podocytes (Mitu and Hirschberg 2008). More specifically in the context of fibrosis, BMP-7 counters the pro-fibrotic effect of TGF-β, reducing extracellular matrix (ECM) formation and contributing to ECM degradation (Li and Tang 2015).

### BMP-7 directly alters autoantibody production and the Th17 response

**Fig. 1:**
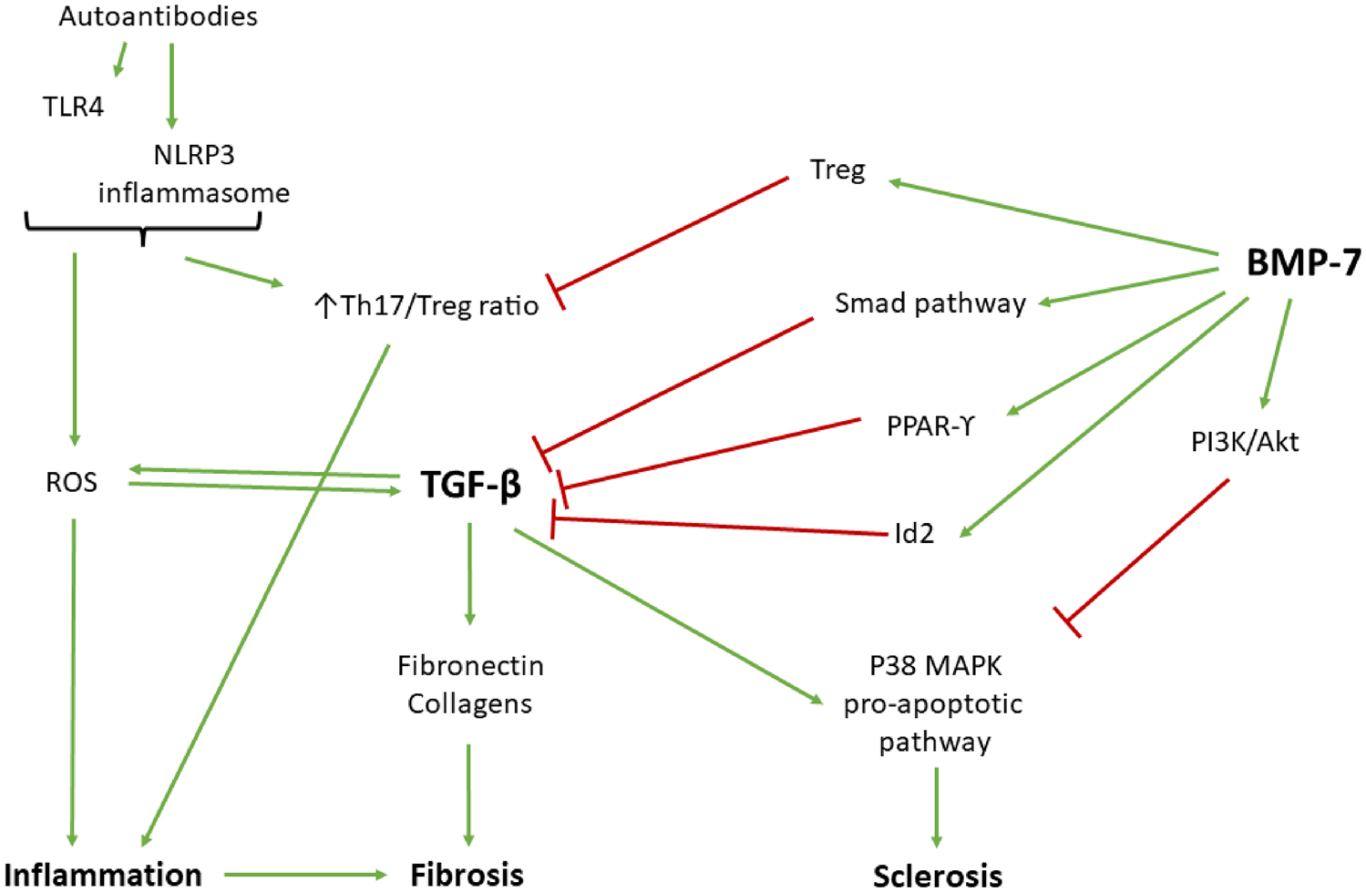
Diagram summarising the pathways regulated by BMP-7 that may be beneficial in the treatment of lupus nephritis. *BMP-7* bone morphogenetic protein-7, *Id2* inhibitor of differentiation 2, *MAPK* mitogen activated protein kinases, *NLRP3* NLR family pyrin domain containing 3, *PI3K* phosphatidylinositol-3-kinase, *PPAR-ϒ* peroxisome proliferator-activated receptor gamma, *ROS* reactive oxygen species, *TGF-β* transforming growth factor-beta, *TLR4* toll-like receptor 4

There is general consensus that autoantibodies directed against double stranded DNA (anti-dsDNA) result in increased reactive oxygen species (ROS) production via TLR4 signalling and activation of the NLRP3 inflammasome that is characterized by an increased Th17/Treg ratio, and which is directly linked to inflammatory and pro-fibrotic processes resulting in loss of organ function in SLE and LN specifically (Zhang et al. 2016; Yuliasih et al. 2019; Paquissi and Abensur 2021). Several beneficial effects of BMP-7 on this pathway have been demonstrated. Firstly, BMP-7 was shown to directly induce apoptosis in human germinal center B-cells (Bollum et al. 2017). Of interest, given the estrogen link to disrupted TGF-β/BMP-7 ratio, the same study also illustrated an opposing effect for TGF-β, likely via its ALK5 receptor. Also, in the context of multiple sclerosis, relapsing patients exhibited overexpression of BMP-2,4,5 but not BMP-7 (Mausner-Fainberg et al. 2013), again suggesting a relative unavailability of BMP-7 in poorer autoimmune disease outcome. Secondly, in osteoarthritis patients with Th17-induced inflammation resulting from exposure to metal debris originating from worn cobalt prosthetic joints, BMP-7 was illustrated to suppress the Th17 response and associated inflammation (Chen et al. 2017). This outcome was likely achieved by the demonstrated ability of BMP-7 to upregulate Treg numbers in tissue (Sconocchia et al. 2021) as summarized in Fig. 1.

### Benefits of BMP-7 demonstrated in models of non-rheumatic renal fibrosis

In kidney disease in general, BMP-7 has been named as an anti-fibrotic prevention strategy in multiple contexts (summarised in Fig. 1). For example, in IgA nephropathy, glomerular fibrosis results from IgA activation of mesangial cells to secrete TNF-α, IL-6 and TGF-β (Julian et al. 1999; Lai et al. 2003) and in patients with IgA nephropathy, significantly reduced levels of glomerular BMP-7 were demonstrated in renal biopsy samples. Furthermore, upon culture of mesangial cells from these patients, addition of BMP-7 significantly reduced fibrosis outcome via activation of peroxisome proliferator-activated receptor-γ (PPAR-γ) and Smad pathways (Chan et al. 2008). Also in HIV-associated neuropathy (HIVAN), higher BMP-7 was associated with less fibrosis and low risk for HIVAN to transition to ESRD, while increased levels of TGF-β1 and TGF-β2 were identified as biomarkers for increased risk of ESRD (Naicker et al. 2021). Moreover, in a model of urethral obstruction-associated renal fibrosis, rapamycin was shown to exert beneficial outcome in terms of fibrosis severity, via reversing the TGF-β/BMP-7 ratio in favour of increased BMP-7 availability (Damião et al. 2007).

Despite the increasing interest in tubulointerstitial germinal cell and fibrosis involvement in disease progression, glomerular injury-associated proteinuria—which results from podocyte injury and/or apoptosis (Lu et al. 2019)—remains an important indicator of acute LN. Therefore, it is important to note that also in this context, benefits for BMP-7 have been demonstrated. For example, glomerular podocyte apoptosis, which is linked to glomerular sclerosis and which is induced by TGF-β activation of the p38 MAPK pro-apoptotic pathway, was opposed by BMP-7 activation of the pro-survival pathway PI3K/Akt (Peters et al. 2006). Similarly, BMP-7 is known to upregulate expression of nestin—a cytoskeletal protein known to protect podocytes from injury in LN (Tian et al. 2020)—to promote regenerative outcome (Zhang et al. 2013; Sun et al. 2023).

In terms of the tubulointerstitium in particular, it is important to consider literature related to diabetic nephropathy, as substantial parallels with LN may be drawn. Diabetic nephropathy was also originally thought to be primarily a glomerular disease, but more recently tubulointerstitial fibrosis is also being recognised as a better indicator of longer term renal functional decline in this context (Phillips and Steadman 2002). Furthermore, in both diabetes and SLE, the transition of tubulointerstitial fibroblasts into myofibroblasts is a major driver of tubulointerstitial fibrosis (Zhang et al. 2012; Lindquist and Mertens 2013). A large body of evidence for a beneficial role for BMP-7 has been established in the context of diabetic nephropathy. For example, in a rodent model of streptozotocin-induced diabetes, BMP-7 upregulated the inhibitor of differentiation (Id2) to reduce tubulointerstitial fibrosis (Xiao et al. 2019). Furthermore, of specific interest to the female bias in LN, in the commonly used *db/db* murine model of diabetes, female sex was linked to a significantly higher TGF-β/BMP-7 mRNA ratio. This was also associated with higher expression of the TGF-β-induced pro-fibrotic cytokine, connective tissue growth factor, which is a known significant role player in myofibroblast activation and deposition of ECM in the progression of fibrosis (Ziller et al. 2020).

### Therapeutic effects of BMP-7 in rheumatic disease models

Pre-clinical data showing a therapeutic effect of BMP-7 in rheumatic disease specifically, is available. Already two decades ago, a study in two genetic models of chronic renal injury and fibrosis—mice deficient in the α3-chain of collagen IV, as well as MRL/MpJ^lpr/lpr^ lupus mice—demonstrated that treatment with recombinant human BMP-7 (rhBMP-7) improved renal function and survival (Zeisberg et al. 2003). Furthermore, rhBMP-7 treatment of (TK-173) human renal fibroblast cells was shown to decrease expression of pro-fibrotic molecules such as fibronectin and collagen I, while upregulating activity of matrix metalloproteinase-2, which is known to remove fibrotic matrix. Perhaps most significantly, this study also provided proof of safety of long-term treatment with BMP-7 at therapeutic level, as no adverse effects were observed during the 4-month treatment intervention.

In terms of a more mechanistic approach, in a triple cell culture simulated model of rheumatoid cachexia, BMP-7 was able to reverse the effect of exposure to human RA patient serum by correcting altered levels of follistatin, myostatin, TGF-β and hepatocyte growth factor, while decreasing abundant collagen IV production (Ollewagen et al. 2022). Similarly, in three dimensional primary cultures of human osteoarthritic chondrocytes, BMP-7 was reported to stimulate proteoglycan synthesis and reduce activity of collagen and aggrecan degrading enzymes (Stöve et al. 2006). Of relevance to the current topic, these beneficial anabolic outcomes is thought to result from BMP-7 antagonism of TGF-β, as TGF-β is implicated as major driver of catabolism in the progression of osteoarthritis (Cui et al. 2016; Long et al. 2016). These studies highlight the potential of BMP-7 to act as a corrective modality in rheumatic conditions.

### Potential for secondary benefits of BMP-7 treatment

Apart from the solid body of evidence in favour of a therapeutic role for BMP-7 in chronic kidney conditions in terms of anti-inflammatory and anti-fibrotic outcome, evidence is also emerging for other effects of BMP-7 which may contribute to patient symptomatic management in SLE and LN.

For example, in a study on 333 SLE patients, pain was reported as one of the major contributors to decreased quality of life in these patients that is not sufficiently addressed by pharmaceutical management of primary disease (Lai et al. 2017). Relevant to this, BMP-7 was reported to play a role in activation of endogenous opioid systems in a rodent study of neuropathic pain (de la Puerta et al. 2019), and may therefore contribute to management of both inflammatory and neuropathic pain in SLE.

Furthermore, obesity and adipose tissue deposition are important factors determining long-term disease prognosis and comorbidity development. For example, patients with SLE werereported to exhibit greater extent of visceral adipose tissue deposition than controls (Li et al. 2019), which—together with the greater perivascular adipose depots reported in rheumatic diseases including SLE (Shi et al. 2022)—predisposes them for cardiovascular complications. As reported for other rheumatic diseases such as RA (Baker et al. 2018), intermuscular adipose tissue deposition is also reported in SLE (Gamboa et al. 2022) and contributes significantly to poorer long-term strength of patients. In addition, given the large number of adipokines secreted within adipose tissue, it is not unexpected that obesity has been identified as a major contributor to sustained pro-inflammatory dysregulation and disease progression in rheumatic disease (Versini et al. 2014), as well as specifically in renal inflammation and fibrosis (Declèves and Sharma 2015). Also in this context, a significant body of literature points to a beneficial role for BMP-7. Firstly, it has been known for some time that BMP-7 reduces appetite, increases weight loss and improves insulin sensitivity in a leptin-independent manner and likely via the mTOR pathway (Townsend et al. 2012). Secondly, BMP-7 was demonstrated to promote differentiation of adipose derived mesenchymal stem cells (AD-MSCs) in brown adipose tissue rather than white adipose tissue both in vitro (Zheng et al. 2014) and in vivo (Townsend et al. 2022), which again confers a more desirable energy expenditure status. Since the majority of studies on BMP-7’s effect on adipose tissue and energy expenditure has been performed in the context of obesity and diabetes, some questions remain unanswered in the SLE context. For example, the potential effect of BMP-7 on intramuscular adipose deposition should be elucidated. Furthermore, given the recent reports of short-term therapeutic benefit of allogeneic AD-MSCs in refractory LN (Ranjbar et al. 2022), the potential of BMP-7 as adjunctive treatment in this context—given its modulatory role on AD-MSC differentiation already mentioned—should be investigated.

## BMP-7-associated drug development: complexities unpacked

### Are cost and market niche obstacles to commercialization?

A comprehensive review of suggested BMP-7-based therapeutic approaches is outside the scope of this review and may be found elsewhere (Lowery et al. 2016; Lowery and Rosen 2018). Briefly, in terms of commercialisation, BMP-7-based drug development has predictably been largely focused on its application in the orthopaedic and dental niches. The reconstructive biotechnology company Stryker Corporation managed to gain “humanitarian device” exemption from FDA approval for their BMP-7 product called OP-1 Putty—a bone graft substitute—for limited orthopaedic application, in 2001. A small-scale randomised clinical trial showed the OP-1 Putty to result in outcome at least comparable to autografts for up to 4 years without evidence of ectopic ossification (Vaccaro et al. 2008). Despite these positive reports, more than a decade later, after changing hands from Stryker to Olympus Biotech, OP-1 Putty was still not FDA approved and commercial interest waned, resulting in discontinuation of the product’s production (Stryker completes sale of OP-1 for use in orthopaedic bone applications to Olympus 2011; Eisner 2014).

The reason for this failure is likely multi-faceted, as pointed out in a systematic review by a UK-based group (Garrison et al. 2007). Here, the authors suggested firstly, that randomized clinical trials (RCTs) on BMPs in general have been underpowered, secondly, that BMP-2 seemed most effective for application in degenerative bone disease and thirdly, that BMP-based therapies were not cost-effective. This disappointing outcome in the orthopaedics niche seemed to have discouraged the commercialisation of BMP-7. However, as is abundantly clear from the extensive body of pre-clinical literature generated that suggest therapeutic potential of BMP-7 in the context of SLE, renal injury and renal fibrosis, BMP-7-based therapies in other contexts should remain a priority. The fact that BMP-7 remains a priority in pre-clinical research, is encouraging.

**Fig. 2:**
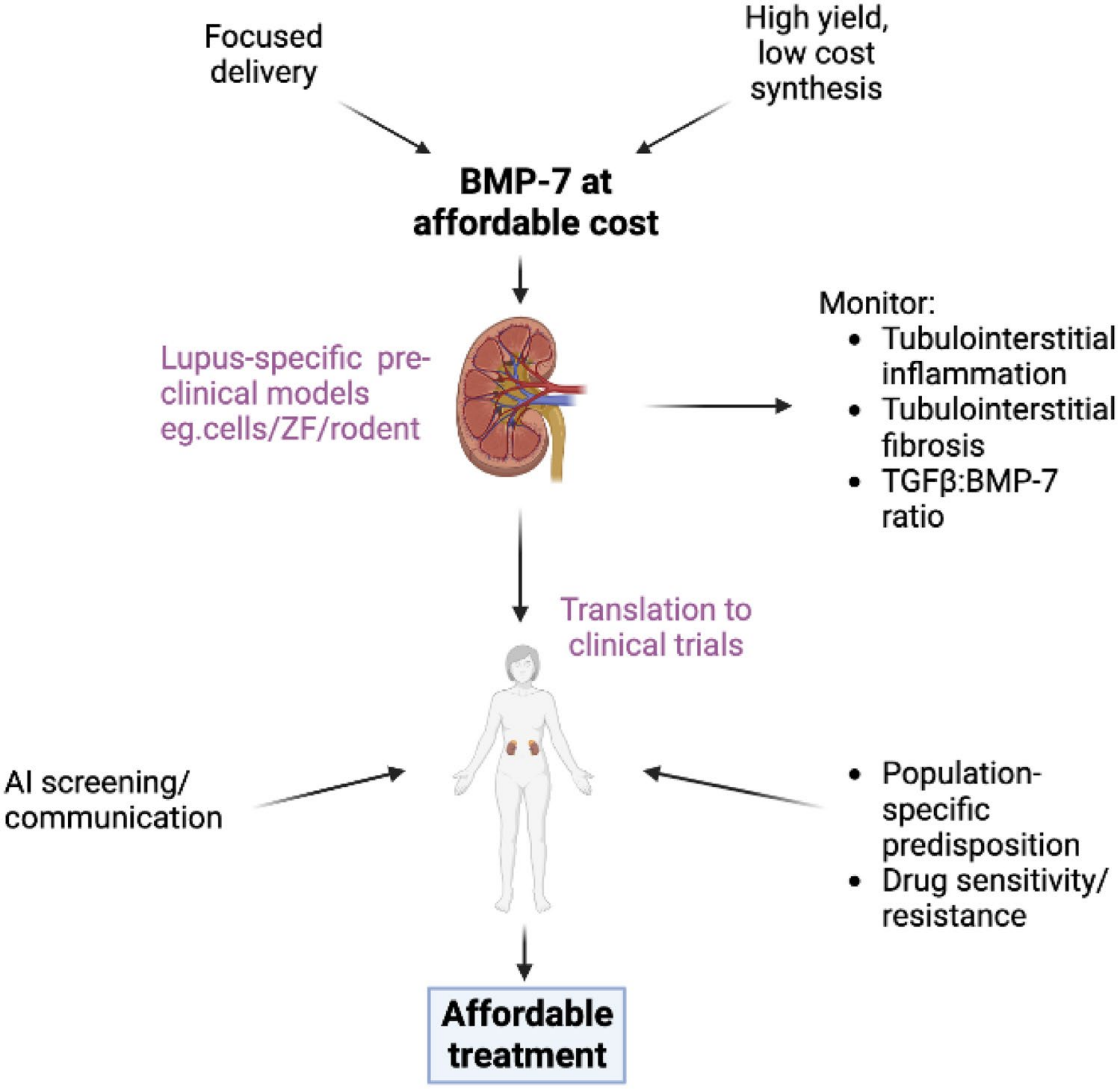
Diagram proposing the potential steps and strategies required for BMP-7 to become an affordable treatment option for lupus nephritis. Investigating mechanisms that allow for focused drug delivery to the region of interest will reduce the amount of BMP-7 protein required for effective treatment. Secondly, in order to reduce the cost of BMP-7 protein itself, better high yield, low-cost synthesis methods are required. Thereafter, pre-clinical disease modelling in appropriate models can elucidate BMP-7 mechanisms of action relative to tubulointerstitial inflammation and fibrosis and contribute to biomarker selection for use in clinical trials. Once adequate monitoring of lupus nephritis is maintained and BMP-7 has been sufficiently tested, research can be translated into the clinical trials arena. Here, artificial intelligence screening and alternative communication methods can reduce the cost of typical clinical trials. Furthermore, patients can be genotyped for patient-specific predisposition and assessed for drug sensitivity/resistance which would alter their treatment interventions. These steps will ultimately lead to more affordable treatment. *AI* artificial intelligence, *ZF* zebrafish

Figure 2 summarises the steps required to move towards affordable BMP-7 for the treatment of lupus nephritis. Cost of treatment remains a significant consideration, especially for conditions such as LN, which has the highest incidence in poorer countries. An encouraging report of symptomatic benefit and general safety and tolerability of BMP-7 injection in patients with osteoarthritis in a 2010 phase I clinical trial provided some proof of potential for—and investor interest in—the “repurposing” of BMP-7 in a rheumatic disease (Hunter et al. 2010) context, but we could not find any more recent reports of clinical trials beyond phase I. The slow progress of BMP-7 through the clinical trial process is likely mainly due to financial considerations. Indeed, a recent paper pointed out although advances in homologous protein expression systems is bringing down the cost of production of potential therapeutic proteins and peptides, that the high cost associated with biologicals may be largely ascribed to the high cost of added expenses, such as clinical trial cost, research and development expenses, patent constraints, marketing, etc. (Puetz and Wurm 2019). Clearly, in order to clear this obstacle, out-the-box thinking is required. One option that is enthusiastically being developed (Askin et al. 2023), is the use of artificial intelligence (AI) to streamline both drug development and clinical trial execution. Although this niche is still in its infancy, the “AI in clinical trial” market is estimated to grow from an estimated 1.5 billion USD in 2022, to 4.8 billion USD by 2027 (AI in clinical trials market 2023). In the context of SLE in particular, AI could make a significant contribution in terms of reaching more patients from diverse populations for participation in clinical trials and retaining them. This is a potential game changer in the context of clinical trial execution in debilitating, but not mainstream, conditions such as SLE.

Likely also due to the high cost reported for BMP-7-based products, more recent research in the context of nephropathy has been focused on more indirect ways to achieve BMP-7 agonism and/or TGF-β antagonism. Most recently, one such product—THR-184, a BMP-7 mimetic—was promoted as an example of the potential of these memetics in the treatment of renal injury (Carlson et al. 2022). However, variable results have been obtained for this product: while it is claimed to have anti-inflammatory, anti-fibrotic and anti-apoptotic effects, as well as ability to reverse epithelial to mesenchymal transition in the context of acute kidney injury, in the paper by Carlson and colleagues, we could not find any peer-reviewed data in support of these claims and a clinical trial failed to show benefit in this context (Himmelfarb et al. 2018). However, more promising advances in biotechnology and drug delivery may increase the effect of BMP-7 mimetics. For example, recent advances in smart nanocarriers, such as those using mesoporous silica nanoparticles (MSNs) which are increasingly used in biomedical applications, may increase drug delivery on demand and to specific target sites (Vallet-Regí et al. 2022), which may reduce the amount of drug required to achieve therapeutic levels. The priority for BMP-7 as a therapeutic is clear from the fact that BMP-7 has already been incorporated into these technologies. For example, a pre-clinical study reported improved osteogenic differentiation of human mesenchymal stem cells after exposure to MSNs laden with BMP-7-derived bone-forming peptide (Luo et al. 2015). Also recently, an injectable silk hydrogel carrier for BMP-7 was described, which could be optimised for desired release kinetics (Townsend et al. 2022), suggesting significant advances in terms of delivery and sustained release of BMP-7 in a more focal manner. These encouraging data warrant further exploration.

### Reported cancer risk potentially misguided

From the previous section, it is clear that therapeutic regenerative and anti-fibrotic levels of BMP-7 carry virtually no risk for undesired off-target effects such as ectopic ossification. However, BMP-7 has been linked to cancer risk. Obviously, such a connotation would be a deal-breaker for any experimental drug. However, in our opinion, these reports may have been somewhat misguided.

For example, most studies suggesting that BMP-7 is linked to adverse outcomes in the context of cancer, base their conclusions on detection of increased BMP-7 levels in tumor tissue (Aoki et al. 2011; Megumi et al. 2012). However, since these studies failed to include assessments of molecules with opposing actions to that of BMP-7, such as TGF-β, these observations may simply suggest a BMP-7 counter-response, rather than a primary driver of cancer pathology. Indeed, more comprehensive studies have indicated a beneficial role for BMP-7 in cancer. For example, in different models of cancer, BMP-7 has been shown to oppose development of cancer metastasis (Na et al. 2009; Shen et al. 2018), via promoting mesenchymal to epithelial transition of cancerous cells, as well as inhibiting telomerase activity in cancer cells, thereby reducing telomere length and inducing cell senescence in cancer cells (Lu et al. 2017; Ning et al. 2019). In the cancer context, the TGF-β opposing activity of BMP-7 is again highlighted as major contributor to beneficial outcome, e.g. by inhibiting TGF-β-driven development of invasive cancer via downregulation of integrin β3 expression (Naber et al. 2012).

Given the complexity of cancer aetiology, it would be unwise to ignore the potential of any growth factor to contribute to cancer risk. However, in the case of BMP-7, a large body of literature in the cancer niche suggests that BMP-7 administration is unlikely to carry significant risk of causing cancer, especially when administered in conditions known to exhibit deficient endogenous BMP-7 levels, such as LN. Existing contradictory studies in the cancer BMP-7 niche highlights the importance of drawing conclusions only from comprehensive analyses, rather than assessment of potential role players in isolation, which may obscure or skew the full picture.

## Conclusion

From our review of the literature, it is abundantly clear that BMP-7 may hold significant promise as a therapeutic modality in SLE and LN. From a mechanistic perspective, benefits of BMP-7 may be much wider than its prominently known antagonism of TGF-β. Despite its potential, no BMP-7-based treatment seems to be in the pipeline yet. Although cost of BMP-7-based treatment development seems to be the major hurdle to implementation, technological advances could contribute to a more affordable solution. This would require biomedical specialists to step outside of their “comfort zone” to embrace AI and nanotechnology solutions in a multidisciplinary approach. Furthermore, given the much wider application potential of BMP-7 in conditions with huge global incidence—such as diabetes and obesity for example—pharmaceutical companies investing in BMP-7 will certainly earn significant return on investment.

## Data Availability

Not appliable, no data sets were generated.
